# *Salix* transect of Europe: patterns in the most abundant chrysomelid beetle (Coleoptera: Chrysomelidae) herbivores of willow from Greece to Arctic Norway

**DOI:** 10.3897/BDJ.4.e10194

**Published:** 2016-09-28

**Authors:** Roy Canty, Enrico Ruzzier, Quentin Cronk, Diana Percy

**Affiliations:** ‡ Natural History Museum, London, United Kingdom; § University of British Columbia, Vancouver, Canada

**Keywords:** Salicophagy, salicivorous insects, Salicaceae, Chrysomelidae, Europe, megatransect

## Abstract

**Background:**

Chrysomelid beetles associated with willow (*Salix* spp.) were surveyed at 41 sites across Europe, from Greece (lat. 38.8 °N) to arctic Norway (lat. 69.7 °N).

**New information:**

In all, 34 willow-associated chrysomelid species were encountered, of which eight were very abundant. The abundant species were: *Crepidodera aurata* Marsham, 1802 at 27 sites, *Phratora vitellinae* (Linnaeus, 1758) at 21 sites, *Galerucella lineola* (Fabricius, 1781) at 19 sites, *Crepidodera fulvicornis* (Fabricius, 1792) at 19 sites, *Plagiodera versicolora* (Laicharting, 1781) at 11 sites, *Crepidodera plutus* (Latreille, 1804) at nine sites, *Chrysomela vigintipunctata* Scopoli, 1763 at nine sites and *Gonioctena pallida* (Linnaeus, 1758) at eight sites. The mean number of willow associated chrysomelid morphospecies at each site was 4.2. Around 20% of the total variance in chrysomelid distribution could be accounted for by latitude, but this is mainly due to distinctive occurrence patterns at the northern and southern parts of the transect. There was a paucity of chrysomelids at Greek sites and a distinctively northern faunal composition at sites north of Poland. Considerable site-to-site variation in colour was noted, except in *G. lineola*, which was chromatically invariant.

## Introduction

Chrysomelidae Latreille, 1802, commonly known as leaf beetles, make up a very large and important major group of phytophagous beetles (Jolivet and Verma 2002). This family is divided in twelve subfamilies﻿ (Haddad and McKenna 2016) and more than twenty tribes but our study mainly focuses on the following three tribes, abundant in our collections: Chrysomelini Latreille, 1802 (Sub. Chrysomelinae Latreille 1802), Galerucini Latreille, 1802 and Alticini Newman, 1834 (Sub. Galerucinae Latreille, 1802). They range from host plant specialists (Jurado-Rivera et al. 2009) to generalist herbivores and many species are recorded feeding on willows.

Willows (*Salix* spp.) are trees and shrubs widespread in N. temperate regions, extending into boreal and arctic habitats. As they are abundant and widespread they form an important food source for specialist and generalist herbivores of all kinds, and are thus ecological “foundation” species (Cronk et al. 2015). Willow feeders include a number of generalist and specialist chrysomelids, which are of great interest for a number of reasons, as set out below.

First, willow-feeding chrysomelids are economically important pests. Willows are a traditional crop for basket making, and more recently they have been extensively planted in both North America and northern Europe﻿ as biomass energy crops. Chrysomelids are potentially destructive pests of such plantations (Larsson and Wirén 1982; Royle and Ostry 1995) with *Phratora vulgatissima* (blue willow beetle), *P. vitellinae* (brassy willow beetle) and *Galerucella lineola* (brown willow beetle) being the major pest species reported.

Secondly, there is considerable variation in the susceptibility of different willows to beetle attack (Hodkinson et al. 1998; Kendall et al. 1996), mirrored by differing feeding preferences of beetles for different willows (Rowell-Rahier 1984; Kelly and Curry 1991b). For instance in Britain, *P. vulgatissima*, while present on most willows, avoids *Salix gmelinii* (syn. *S. burjatica*, *S. dasyclados* Wimm.) and Salix × mollissima (*S. triandra* × *S. viminalis*) (Sage and Tucker 1998). In a study which tested preference by *P. vulgatissima* for the segregating progeny of the cross *S. gmelinii* × *S. viminalis*, a great variation in herbivore performance (survival and oviposition success) was found (Torp et al. 2013). Kelly and Curry 1991b have shown that the resistance of *S. gmelinii* to herbivory by *P. vulgatissima* is likely due to the high amounts of the toxic phenolglycoside (salicylate) salicortin in the plant. Phenolglycoside occurrence varies greatly in willows, occurring in *S. nigricans*, *S. purpurea* and *S. fragilis* but absent in *S. alba*, *S. caprea* and *S. cinerea* (Rowell-Rahier 1984) and it has been suggested that the presence of toxic phenolglycosides promotes host specificity among herbivores (Rowell-Rahier 1984), while deterring generalist such as *P. vulgatissima* and *G. lineola* (Kendall et al. 1996). All this suggests that there is a complex co-evolutionary context existing between willows and their herbivorous beetles, mediated by plant biochemistry.

Thirdly, willow-feeding chrysomelids have a remarkable chemical ecology in which the larvae of the beetles use plant-derived chemicals for defence (Boland 2015; Pasteels et al. 1988). It is postulated that defence in these beetles was originally through purely endogenously-synthesized chemicals, but adaptation to feeding on highly toxic willow hosts facilitated a transfer to plant-derived molecules (Pasteels et al. 1990). When attacked, chrysomelid larvae discharge toxic droplets from glandular reservoirs on their backs. These glands have been called “bioreactors” (Boland 2015) as they perform final steps of toxin synthesis from plant chemicals trafficked into the glands by an intricate molecular transport system. For instance *P. vitellinae* secretes a copious amount of salicylaldehyde (Pasteels et al. 1982; Pasteels and Gregoire 1984). Salicylaldehyde is produced by hydrolysis of plant derived salicin to salicyl alcohol, followed by oxidation to salicyladehyde (Hilker and Schulz 1994). Another species, *Chrysomela lapponica*, shows population variation in their chemistry. Populations associated with salicin-poor willows or birches do not produce salicylaldehyde whereas populations associated with salicin-rich willows do (Gross and Hilker 1994; Geiselhardt et al. 2015). Predators, both carnivorous sawfly larvae (Hymenoptera: *Tenthredo*; Pasteels and Gregoire 1984) and ants (Hymenoptera: *Formica*; Zvereva et al. 2016) are initially repelled by the larval secretion but can both overcome the repulsion with experience, indicating that the defence may be most effective when predation levels are relatively low. It is not only the larvae that are chemically defended as some species, for instance *P. vitellinae* sequester salicin in their eggs which is an effective deterrent to ant predation (Pasteels et al. 1986). However, defence is not the only effect of these secretions as they also regulate conspecific and interspecific intergenerational competition by deterring feeding and oviposition by adults of the same species as well as other chrysomelid species (Hilker 1989). This anti-competitive effect may be as important as the defence role, if not more important.

Fourthly, the willow-feeding chrysomelids form host races with distinctive host specificity. The example was given above of substantial differences in biochemistry between populations of *C. lapponica* (particularly the ability to use salicin as a substrate). This is not the only example of recent evolution in the group. Particular interest attaches to *Lochmaea capreae* (Linnaeus, 1758), which like *C. lapponica* has willow and birch associated populations, but in this case they are sympatric (Soudi et al. 2015). The host-specific populations of *L. capreae* have been shown to have a genetic basis and to be true host races (Soudi et al. 2016). An intriguing example of active evolution is provided by *Plagiodera versicolora* (Laicharting, 1781), in which populations are under selection either for feeding exclusively on new leaves (gourmet populations) or on all leaves (no preference populations) (Utsumi et al. 2012, Utsumi et al. 2009). In this instance the feeding preference feeds back via plant response to the herbivory to have a profound effect on the willow-associated arthropod community composition and dynamics. For instance gourmet feeding by chrysomelids resulted in more aphids (Utsumi 2015).

Fifthly, the willow-feeding chrysomelids are prone to outbreaks and thus have an interesting and dynamic population biology. For instance a study of *P. vulgatissima* on *Salix viminalis* in Ireland (Kelly and Curry 1991a) showed a variation in successive years from maximal mean densities of 308 adults per tree to 72 adults per tree the following year. A study of the same species found that beetle density was lower in mixed species willow stands than in monocultures (Peacock and Herrick 2000). Chrysomelid populations are regulated by predators including heteropteran bugs such as *Anthocoris* and parasitoids. Herbivory by *P. vulgatissima* has been shown to attract *Anthocoris* (Lehrman et al. 2013). The parasitoid wasp, *Perilitus brevicollis* Haliday 1835, also attacks *Phratora vulgatissima*. However, somewhat paradoxically, control is limited at high beetle densities, as at high densities beetles become smaller, which causes parasitoid survival to decrease (Stenberg 2015).

Sixthly, it should be noted that many willow-feeding chrysomelids have highly temperature dependent development and thus should be highly responsive to interannual climatic variation and, ultimately, to climate change. Perhaps related to this, chrysomelids are known to have distinctive distribution patterns within Europe (Schmitt and Rönn 2011). Kutcherov (2015) has shown that *Chrysomela vigintipunctata* requires 275.5 degree days (DD) above a threshold of 9.0 °C for egg to adult development. In cold weather the adults appear later and are larger (as development has been slower). In warmer weather adults appear sooner and are smaller (having developed fast). Changes in beetle distribution, phenology or size with changing temperature may in turn have knock-on effects on other willow-associated arthropod communities, and perhaps thereby on whole ecosystems.

Most studies involving willow-feeding chrysomelids are specific to a single locality or geographical region. We wished to determine the most abundant species of willow-associated chrysomelids over a wide geographical range and to assess their patterns of occurrence and co-occurrence, and their population variability as part of a broader study on willow communities across Europe. Therefore chrysomelid beetles were collected by one of us (ER) from 41 willow stands over a north-south megatransect from Greece to Arctic Norway. This megatransect has been described previously (Cronk et al. 2015).

## Materials and Methods

### Site selection and details

Full details of the sites and their selection have been given previously (Cronk et al. 2015). Briefly the route from Greece to Arctic Norway was driven in 2015, stopping approximately every 100km to locate and sample a stand of willows (*Salix* spp.) (Table 1).

### Collecting methods

**Figure 1.**
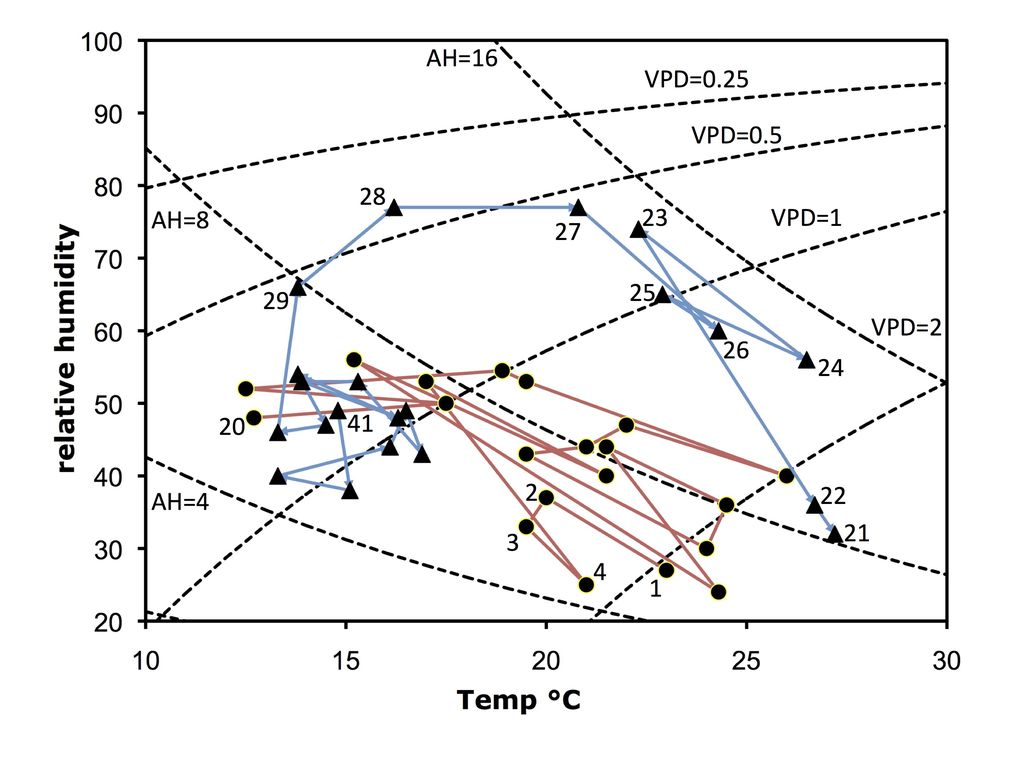
Collecting conditions (temperature and relative humidity) at the sites (data plotted from Table 2). In this graph lines of constant absolute humidity (AH; g/m^3^) and vapour pressure deficit (VPD; kPa) are plotted as dashed lines. VPD is a measure of the drying power of the air. Circles (red line) mark collection localities 1-20 (April 2015) while the triangles (blue line) mark sites 21-41 (June 2015). Note that the environmental conditions during collection are very similar between Central Europe (sites 18-20) in April and Arctic Europe (sites 30-41) in June.

Willow associated beetles were collected at every site. A sweep net was used with an attempt to sample from all the taxa of willows present at a site. Willows commonest at a site were sampled more. Sampling duration was approximately 1 hour per site. An attempt was made to separate collections from each species of willow, but as field identification of willows is often difficult and complicated by hybridization this was not always possible. For the purposes of this paper all samples at a site are pooled. The willows at each site and voucher herbarium specimens are given elsewhere (Cronk et al. 2015). Beetle samples were immediately transferred, in the field, into tubes containing 70% alcohol. Alcohol preserved material was then kept at ambient temperature and transferred to the NHM (London) for subsequent sorting. As collecting efficiency may be influenced by environmental conditions the time of day, relative humidity (rH) and temperature (t°C) were also recorded for each site (Table 2; Fig. 1). Relative humidity and temperature were recorded using a Hyelec MS6508 thermohygrometer.

### Specimen examination and analysis

Specimens from each locality were sorted into broad morphospecies, identified and counted. Identifications were made by RC. Most morphospecies likely correspond to biological species. The following works and resources were consulted for the identification of taxa: Hubble (2012); Borowiec (2013); Warchałowski (2003); Warchałowski (2010); Lompe (2002); Watford Coleoptera Group (2016); and the species list of Volf et al. (2015). Some of the most abundant species (*Crepidodera aurata* Marsham, 1802, *C. fulvicornis* (Fabricius, 1792), *C. plutus* (Latreille, 1804), *Phratora vitellinae* (Linnaeus, 1758), *Galerucella lineola* (Fabricius, 1781), *Plagiodera versicolora* (Laicharting, 1781), *Chrysomela vigintipunctata* Scopoli, 1763 and *Gonioctena pallida* (Linnaeus, 1758)) were subsampled (one to three individuals per sample from 6 samples per species) from selected localities for imaging and measurement. Measurements were performed using a Zeiss Stemi DV4 dissecting scope and a Minitool miniature measuring scale with a 5mm range calibrated to 0.1mm. Colours were determined by matching to the standard RHS colour chart (RHS 2001). Colour codes were translated to colour names using standard practice (UPOV 2013). Photographs were taken using a Canon EOS 700D, viewing through a Leica MZ12.5 stereomicroscope. Photos were taken via a Dell computer using the Canon EOS 700D Utility Remote Live View programme to take several photos of each specimen at different focus distances. These photos were then combined together to form a fully focused image using the focus stacking software Helicon Focus (version 5.3).

### Data Analysis

The inter-site latitudinal variation in occurrence of the eight commonest species (Table 4) was examined using Canonical Principal Components Analysis (Redundancy Analysis), with latitude as the explanatory variable. The beetle matrix of counts of individuals (Table 4) was square root transformed to normalise. The beetle matrix was used as the response matrix. Redundancy analysis was performed using the Java package Ginkgo in the software suite B-VegAna (Font Castell 2006).

## Results

### Species encountered and their relative abundance

The list of species encountered is given in Table 3. The sites along the transect yielded 34 morphospecies of willow-associated chrysomelid. The most widespread and abundant of these was *C. aurata*, which occured at 27 out of 41 sites and >267 individuals were captured in our samples. In all, eight morphospecies were common, occurring at eight or more sites and in considerable abundance (Table 3). The remaining 26 morphospecies were comparatively sparsely distributed with 11 being found at a single site only. The eight common species contributed 1164 counted individuals in our samples. The remaining 26 morphospecies contributed only a further 128 counted individuals (Table 3). Most of the species are known willow-feeders. However, some species taken from willow are commonly recorded as feeding exclusively on other types of plant (Böhme 2001): *Donacia aquatica* (*Carex* spp.), *D. simplex* (*Sparganium* spp.) and *Chrysolina graminis* (Asteraceae). Nevertheless they are included here as willow-associated, and examples of beetles that may be taken as by-catch when sampling willows. Site descriptions (Cronk et al. 2015) for sites 29 and 38, where *Donacia* is present, indicate their suitability (as wetland sites) for these species.

Chrysomelids were rare in Greece and were absent from two Greek sites sampled (2 & 5). However, they were generally abundant at all other sites (from Bulgaria to Norway) (Table 4). The mean number of captured and counted individuals at Greek sites was 2.2 with a range of 0-7. For the remaining sites the mean was 35.6 (range 5-90) (Table 4).

In terms of number of morphospecies per site, Greek sites had an average of 1 species (range 0-2) and the other sites an average of 4.7 species (range 2-12) (Table 3). All sites together had an average number of species per site of 4.2.

### Geographical Patterns in the commonest species

The commonest species and their site distributions are detailed in Table 4. The species showed clear evidence of geographical patterning (Table 4). Of the eight abundant species, three were very widespread along the transect (*C. aurata*, *P. vitellinae* and *G. lineola*). Two had northern-biased distributions (*C. fulvicornis* and *Gonioctena pallida*) and three southern-biased distributions (*P. versicolora, C. vigintipunctata* and *C. plutus*).

### Correlations with latitude

Redundancy analysis showed that variation in occurrence of chrysomelids (common species) was, as expected, highly correlated with latitude. Latitude was able to explain 23.2% of the total variance in the beetle matrix. When the latitude input order was randomized multiple times, latitude was only able to explain around 2% of the variance by chance alone (mean=2.26%, standard deviation = 0.71). However this correlation with latitude is mainly due to (1) the paucity chrysomelids at the southernmost sites (Greece) and (2) the difference between a distinctly boreal chrysomelid fauna north of Poland contrasting with a rather homogeneous central European fauna from Bulgaria to Poland (sites 6 to 23). When sites 6 to 23 are analyzed separately there is little association with latitude (6.8%) and this is not much better than random (random: 3.66%, SD 1.36).

### Morphological Variation

**Figure 2.**
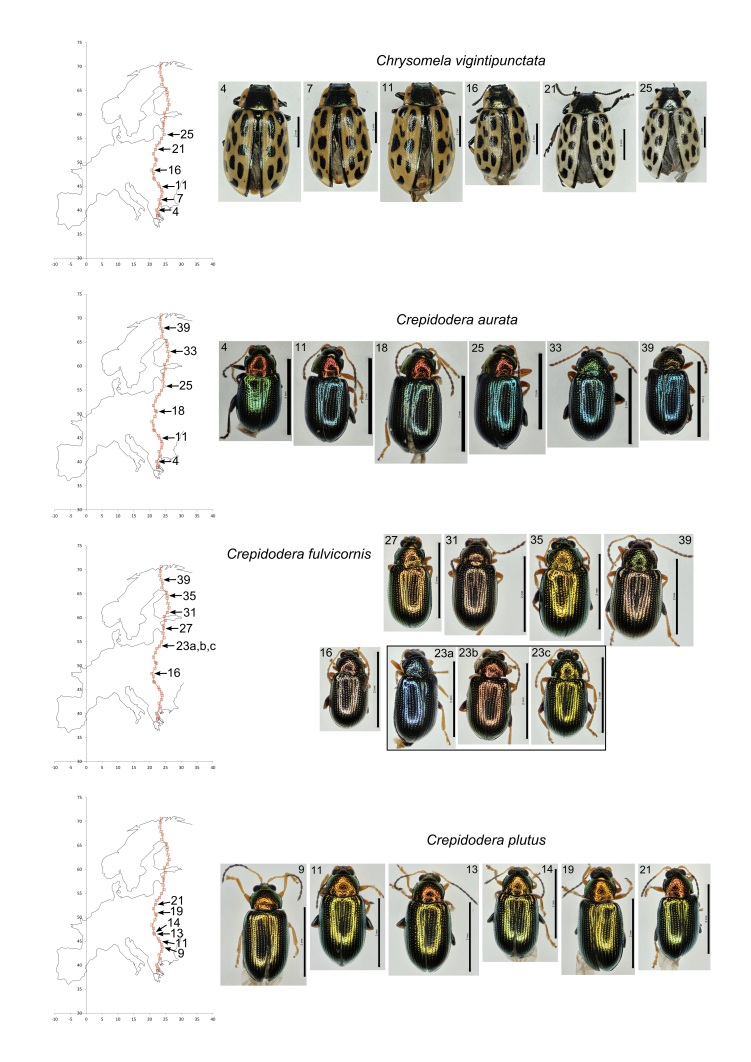
Images of representative examples of common species from different populations. *Chrysomela vigintipunctata, Crepidodera aurata, C. fulvicornis, C. plutus*. Populations are referred to a map (left). Scale bars = 1 mm.

**Figure 3.**
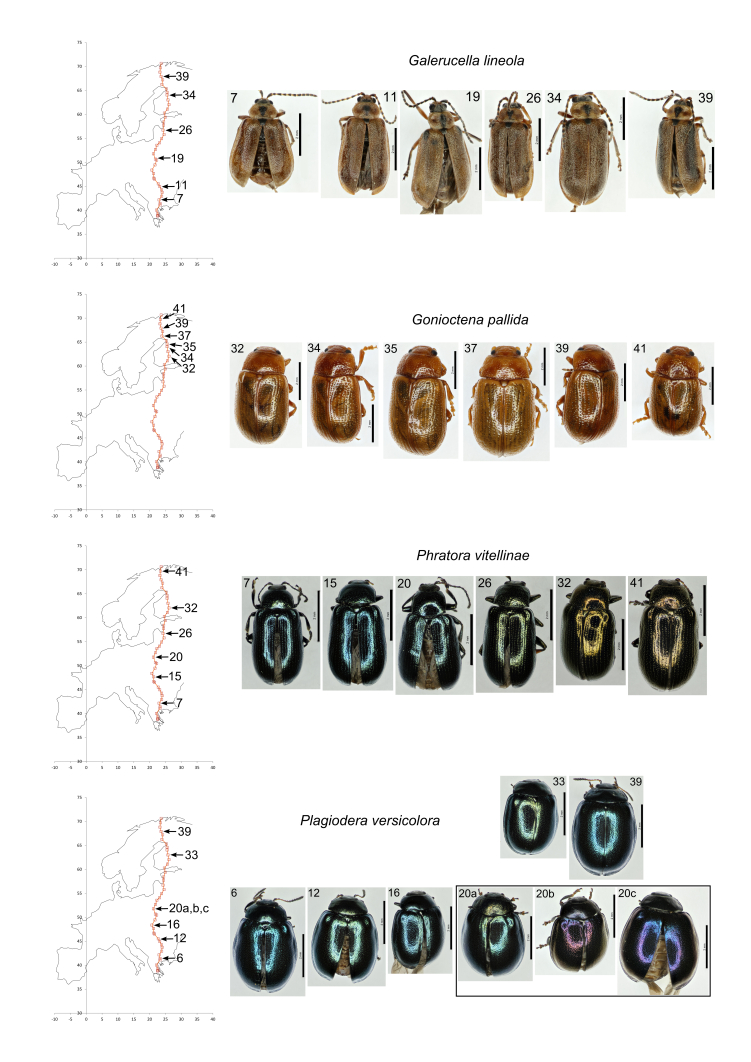
Images of representative examples of common species from different populations. *Galerucella lineola*, *Gonioctena pallida*, *Phratora vitellinae*, *Plagiodera versicolora*. Populations are referred to a map (left). Scale bars = 1 mm.

We noted considerable variation in colour and size of the common beetles from population to population but within populations they tended to be fairly homogeneous. All the common species displayed great chromatic variation (Table 5; Fig. 2; Fig. 3) with the exception of *G. lineola*. In this species no variation in colour was detectable by the human eye. Species also differed in their size variation: most were quite variable between populations but the three species of *Crepidodera* were comparatively invariant in size (Table 5).

## Discussion

### A single-year, time-limited snapshot

The distribution and abundance of chrysomelids does not just vary geographically. These beetles are well known for temporal variation, both phenological (timing of appearance), population build-up during a year and interannual (year to year) variation driven by episodic outbreaks and population control by parasites and predators. The variation between willow stands, and across Europe will reflect both spatial and temporal patterns. Nevertheless, our “snapshot” of variation gives a clear idea of the variation across Europe to be encountered in a particular year. It also provides the possibility for follow-up specifically to quantify temporal variation. Another advantage of collecting along a geographically wide megatransect is that a full picture of morphological variation within a species is gained (as summarized in Table 5). Biogeographical work in central Europe (Schmitt and Rönn 2011) characterized *Crepidodera fulvicornis* as "widely distributed", while *Gonioctena pallida* and *Phratora vulgatissima* were characterized as "southern", on the basis of 63,000 records. The differences in biogeographical pattern reported here could be due to the "snapshot effect" or simply to the different (more easterly) region being examined. Further work will be needed to distinguish these two hypotheses.

### Potential distributional breakpoints

It is clear that our sampling reveals a considerable difference between Greece and Bulgaria. This may reflect the comparative rarity of willows in the strongly anthropogenically disturbed and dry Mediterranean climate of Greece, which would deny willow-associated beetles the ready access to this food-plant resource that they have over the rest of Europe. Another possible explanation is that the paucity of *Salix*-associated chrysomelids in Greece in 2015 is the consequence of phenology or interannual variation (the spring was noted to have been exceptionally warm in Greece in 2015).

Another potential distributional breakpoint we note is around site 23 (northern Poland) which appears to mark a division between the southern-biased common species which end around here (at sites 20-25) and the northern-biased species *C. fulvicornis* which comes in strongly at site 25 (admittedly with southern outliers to site 11). The other northern-biased species, *Gonioctena pallida*, does not fit the pattern so well, coming in at site 32 (Finland). However this may be due to our late timing of collection with respect to what is clearly a more cryophilous beetle. Generally, the apparent transition point in northern Poland may reflect a genuine biogeographical shift or may simply reflect the particular circumstances of phenology and collection time.

Although this transect was north-south in orientation, the effect of east-west biogeographical boundaries can be seen in the comparative rarity of *P. vulgatissima* (3 sites only). This beetle is sometimes stated to be the commonest willow-associated chrysomelid in Europe (as also implied by its Linnaean epithet) so it might appear odd that it was not more abundant in our samples. However it is a species primarily of NW Europe, being particularly abundant in Sweden and Germany westwards to the UK and Norway. Our transect goes through the eastern edge of its range so the comparative rarity in our samples in not surprising.

## Figures and Tables

**Table 1.** Site details. Further information can be found in Cronk et al. (2015).

**SITE**#	**Country**	**Lat N**	**Long E**	**Alt (m)**
1	Greece	38.80007	22.4629	37
2	Greece	38.902	22.31015	33
3	Greece	39.306694	22.528323	177
4	Greece	40.032685	22.175437	534
5	Greece	41.113317	23.273893	31
6	Bulgaria	41.412468	23.318609	90
7	Bulgaria	42.165622	22.998141	392
8	Bulgaria	42.923989	23.810563	339
9	Bulgaria	43.739343	23.966755	35
10	Romania	44.260343	23.786781	81
11	Romania	44.961981	23.190337	172
12	Romania	45.510676	22.737225	556
13	Romania	46.518504	21.512839	102
14	Hungary	46.700744	21.31268	94
15	Hungary	47.665648	21.261768	91
16	Hungary	48.374291	20.725264	148
17	Poland	49.463447	21.697255	385
18	Poland	50.470234	22.238372	157
19	Poland	50.673994	21.823391	141
20	Poland	51.775039	21.1971	101
21	Poland	52.69398	21.8529	96
22	Poland	53.55483	22.30299	128
23	Poland	54.06943	23.11745	137
24	Lithuania	54.92583	23.7742	28
25	Lithuania	55.79557	24.56678	62
26	Latvia	56.71141	24.25162	23
27	Latvia	57.74963	24.4023	7
28	Estonia	58.42257	24.44063	18
29	Estonia	59.40289	24.93577	48
30	Finland	60.27299	24.65843	33
31	Finland	61.09965	25.6282	84
32	Finland	62.04962	26.12369	174
33	Finland	63.01589	25.80457	139
34	Finland	64.05074	25.52664	91
35	Finland	64.61287	25.53805	58
36	Finland	65.32835	25.29175	1
37	Finland	66.24947	23.8945	51
38	Finland	67.21253	24.12629	160
39	Finland	67.91183	23.63411	233
40	Norway	68.8138	23.26658	374
41	Norway	69.72487	23.40581	289

**Table 2.** Date, time and environmental conditions at the start of collection.

**SITE**	**temp C**	**humidity** %	**time**	**date**
1	23	27	13.35	21-iv-2015
2	20	37	16.4	21-iv-2015
3	19.5	33	12.1	22-iv-2015
4	21	25	17.05	22-iv-2015
5	17	53	12.25	23-iv-2015
6	21.5	40	17	23-iv-2015
7	15.2	56	10.3	24-iv-2015
8	24.3	24	16.3	24-iv-2015
9	21.5	44	19.05	24-iv-2015
10	24.5	36	13.05	25-iv-2015
11	24	30	16.3	25-iv-2015
12	19.5	43	10.25	26-iv-2015
13	21	44	18.05	26-iv-2015
14	22	47	10.3	27-iv-2015
15	26	40	16.3	27-iv-2015
16	19.5	53	11.5	28-iv-2015
17	18.9	54.5	18	28-iv-2015
18	12.5	52	12	29-iv-2015
19	17.5	50	15	29-iv-2015
20	12.7	48	9	30-iv-2015
21	27.2	32	12.3	12-vi-2015
22	26.7	36	17.15	12-vi-2015
23	22.3	74	10	13-vi-2015
24	26.5	56	14.45	13-vi-2015
25	22.9	65	19.4	13-vi-2015
26	24.3	60	10	14-vi-2015
27	20.8	77	15.45	14-vi-2015
28	16.2	77	8.3	15-vi-2015
29	13.8	66	13.4	15-vi-2015
30	13.3	46	10.3	16-vi-2015
31	14.5	47	16	16-vi-2015
32	13.8	54	10.45	17-vi-2015
33	16.3	48	15	17-vi-2015
34	13.9	53	19	17-vi-2015
35	15.3	53	12	18-vi-2015
36	16.9	43	16	18-vi-2015
37	16.5	49	10.15	19-vi-2015
38	16.1	44	14.3	19-vi-2015
39	13.3	40	18.15	19-vi-2015
40	15.1	38	11.3	20-vi-2015
41	14.8	49	15.45	20-vi-2015

**Table 3.** Species recorded, in order of number of sites. The first 8 species are the most widespread and have sufficient representation to be classified into wide, northern and southern occurrence tendencies.

**SPECIES**	**Number of Sites (S)**	**No. of Individuals (N)**	**Abundance index (NxS)**	**Site Range**
*Crepidodera aurata* Marsham, 1802	27	>267	7209	3 - 39 [Wide]
*Phratora vitellinae* (Linnaeus, 1758)	21	>215	4515	7 - 41 [Wide]
*Crepidodera fulvicornis* (Fabricius, 1792)	19	191	3629	(11-)23-39 [Northern]
*Galerucella lineola* (Fabricius, 1781)	19	>267	5073	11 - 39 [Wide]
*Plagiodera versicolora* (Laicharting, 1781)	11	43	473	6-20(-39) [Southern]
*Chrysomela vigintipunctata* Scopoli, 1763	9	34	306	4 - 25 [Southern]
*Crepidodera plutus* (Latreille, 1804)	9	>57	513	9 - 23 [Southern]
*Gonioctena pallida* (Linnaeus, 1758)	8	>90	720	32 - 41 [Northern]
*Altica* sp.	4	7	28	6,8,22,23
*Chrysomela populi* Linnaeus, 1758	3	5	15	12,13,17
*Crepidodera aurea* (Geoffroy, 1785)	3	8	24	12,30,32
*Cryptocephalus* sp.	3	11	33	3,6,24
*Phratora vulgatissima* (Linnaeus, 1758)	3	15	45	15,18,39
*Agelastica alni* (Linnaeus, 1758)	2	2	4	28,30
*Chaetocnema picipes* Stephens, 1831	2	2	4	11,18
*Chaetocnema* sp.	2	2	4	11,23
*Cryptocephalus decemaculatus* (Linnaeus, 1758)	2	2	4	25,28
*Dibolia* sp.	2	3	6	6,7
*Gonioctena linnaeana* Schrank, 1781	2	4	8	38,39
*Gonioctena viminalis* (Linnaeus, 1758)	2	14	28	33,37
*Lochmaea caprea* (Linnaeus, 1758)	2	9	18	25,26
*Longitarsus* sp.	2	2	4	11,27
*Smaragdina salicina* (Scopoli, 1763)	2	2	4	12,13
*Bromius obscurus* (Linnaeus, 1758)	1	4	4	33
*Chrysolina fastuosa* Scopoli, 1763	1	2	2	1
*Chrysolina graminis* Linnaeus, 1758	1	2	2	37
*Cryptocephalus sexpunctatus* (Linnaeus, 1758)	1	5	5	11
*Cryptocephalus exiguus* Schneider, 1792	1	3	3	24
*Donacia aquatica* Kunze, 1818	1	1	1	38
*Donacia simplex* Fabricius, 1775	1	1	1	29
*Gonioctena nivosa* (Suffrian, 1851)	1	1	1	33
*Lytharia salicariae* (Paykull, 1800)	1	2	2	26
*Phratora laticollis* Suffrian, 1851	1	18	18	11
*Smaragdina flavicollis* Charpentier, 1825	1	1	1	28

**Table 4.** Abundance of common species at sites. Counts of individuals are given for all samples. Abbreviations: *Ch. vig.* = *Chrysomela vigintipunctata* Scopoli, 1763; *Cr. aura.* = *Crepidodera aurata* Marsham, 1802; *Cr. fulv.* = *Crepidodera fulvicornis* (Fabricius, 1792); *Cr. plutus* = *Crepidodera plutus* (Latreille, 1804); *G. lineo.* = *Galerucella lineola* (Fabricius, 1781); *Gonio. pal.* = *Gonioctena pallida* (Linnaeus, 1758); *Ph. vitel.* = *Phratora vitellinae* (Linnaeus, 1758); *Pl. vers.* = *Plagiodera versicolora* (Laicharting, 1781); Tot (com) = Total individuals at sites (common species); Tot (all) = Total individuals at sites (all species); N. spp. = number of chrysomelid species at sites. Counts marked > indicate that not all individuals were counted.

**Site**	***Ch. vig.***	***Cr. aura.***	***Cr. fulv.***	***Cr. plutus***	***G. lineo.***	***Gonio. pal.***	***Ph. vit.***	***Pl. vers.***	**Tot. (com)**	**Tot. (all)**	**N. spp**
1									0	1	1
2									0	0	0
3		2							2	3	2
4	1	6							7	7	2
5									0	0	0
6	1	1						11	13	18	6
7	2	4					16	5	27	29	5
8		30							30	31	2
9				9				1	10	10	2
10		2		4				3	9	9	3
11	4	32	1	3	9		2	1	52	78	12
12		15		3				3	21	26	6
13	1	6		3					10	13	5
14		12		7	1				20	20	3
15		6					2		8	10	3
16	10	22	1		1		1	6	41	41	6
17	3	>40					20		63	64	4
18		>20			9			1	30	38	5
19		4	4	>20	5				33	33	4
20		2			1		3	7	13	13	4
21	1			7					8	8	2
22		4							4	5	2
23			26	1	7				34	39	6
24									0	10	2
25	11	15	7				1		34	36	6
26		1	11		9		>20		41	51	6
27		3	4				3		10	11	4
28		12	19				24		55	58	6
29		6	1		3		1		11	13	6
30		2	17		8		3		30	32	6
31			12		3		19		34	34	3
32			16		22	1	26		65	70	5
33		1	8		8		9	3	29	39	8
34			11		25	1	5		42	42	4
35			34		>50	1	1		86	86	4
36		2	10		>40	1	6		59	59	5
37		2	5		5	6	18		36	47	7
38		10	1		>50		27		88	90	6
39		5	3		11	10		2	31	40	7
40						>30			30	30	1
41						40	8		48	48	2
								TOTS:	1164	1292	

**Table 5.** Measurements of six to eight representative individuals of the common Chrysomelids (one to three per site) chosen to show variation.

**Species**	**Sites**	**Elytral Colour on scored individuals**	**Main elytral colours (sites)**	**Elytral length (mm)**	**Elytral width at shoulder (mm)**	**Pronotal length (mm)**	**Pronotal width at base (mm)**
*Chrysomela vigintipunctata*	4, 7, 11, 16, 21, 25	161B, 162B, 162C, 161C, 155C, 155C	Light yellow brown (4, 7, 11, 16); white (21, 25)	5.3-6.8	3.0-3.7	1.4-1.5	2.7-3.2
*Crepidodera aurata*	4, 11, 18, 25, 33, 39	135B, 118C, 119B, 118C, 118B, 111B	dark green (4); light green blue (11, 25); grey blue (18); green blue (33, 39)	2.0-2.2	1.2-1.2	0.5-0.6	1.0-1.1
*Phratora vitellinae*	7, 15, 20, 26, 32, 41	111A, 111A, 111B, 137B, N144A, 146D	green blue (7, 15, 20); brown green (26, 41); dark green (32)	3.5-3.9	1.9-2.4	1-1.1	1.5-2.0
*Plagiodera versicolora*	6, 12, 16, 20, 20, 20, 33, 39	111B, 118B, 113B, 113B, N80B, N87B, 120B, 113B	green blue (6, 12, 16, 20, 39); violet (20); light blue green (33)	2.9-3.9	2.3-2.5	0.9-0.9	1.9-2.2
*Crepidodera fulvicornis*	16, 23, 23, 23, 27, 31, 35, 39	137B, 104B, 175D, N144B, 144B, 143B, N144B, 143C	brown green (16); medium blue (23); medium brown (23); light green (23,27, 35); dark green (31, 39)	1.7-2.2	1-1.3	0.5-0.6	0.8-1.1
*Galerucella lineola*	7, 11, 19, 26, 34, 39	165A, 165A, 165A, 165A, 165A, 165A	medium brown (all)	3.5-4.4	1.9-2.2	0.8-0.9	1.4-1.5
*Crepidodera plutus*	9, 11, 13, 14, 19, 21	N144A, N144B, N144B, 141B, N144B, 141A	light green (9,11, 13, 19); dark green (14, 21)	2.1-2.4	1.2-1.3	0.5-0.6	1.0-1.0
*Gonioctena pallida*	32, 34, 35, 37, 39, 41	165B, 165B, 165B, N167A, N167B, 165B	yellow brown (all)	3.6-4.8	2.8-3.0	1.3-1.3	2.6-2.8
